# First-Day Glycemic Exposure and 28-Day Mortality in the ICU: A Multicenter Cohort Study

**DOI:** 10.21203/rs.3.rs-9032328/v1

**Published:** 2026-03-08

**Authors:** Joab O. Odera, Betsabe Blas, Julie Cha, Aisha Montgomery, Alice A. Ojwang, Sepiso Masenga, Elizabeth O. Odera, Nosayaba Osazuwa-Peters, Ananya Yalamanchi, David Han, Antentor O. Hinton

**Affiliations:** Duke University School of Medicine; Baylor College of Medicine; Georgia Institute of Technology; Premier Inc.; Diabesity Nutrition Clinic Inc.,; Vanderbilt University School of Medicine; Iris Laboratories Ltd.; Duke University School of Medicine; Memorial Hermann Texas Medical Center; University of Texas at San Antonio; Vanderbilt University School of Medicine

**Keywords:** glycemic exposure, critical illness, hyperglycemia, survival analysis, artificial intelligence

## Abstract

**Importance:**

Early glycemic exposure in the ICU is common and clinically modifiable, yet bedside assessment often relies on single glucose values rather than exposure-aware metrics. Interpretable, first-day prediction may support individualized glycemic targets and early intervention.

**Objective:**

To examine the association between first-day time-weighted average glucose (TWAG) and 28-day mortality, and to evaluate GlucoSurvAI, an interpretable ensemble model for first-day risk stratification.

**Design, Setting, and Participants:**

Retrospective cohort study using electornic health records from 13 U.S. hospitals. Among 18,868 adult ICU encounters, 8,048 patients from 7 U.S. hospitals met inclusion criteria (≥1 glucose value and hospital length of stay ≥24 hours).

**Exposures:**

First-day glycemic exposure summarized as TWAG, categorized as <100, 100–139 (reference), 140–179, and ≥180 mg/dL. Prespecified covariates included diabetes/prediabetes, first-day insulin and glucose, corticosteroids, vasopressors, shock, cancer, glucose-monitoring intensity, and clinical site.

**Main Outcome and Measures:**

Primary outcome: 28-day all-cause mortality. Associations were estimated with multivariable Cox models (adjusted hazard ratios [aHRs], 95% CIs). GlucoSurvAI performance was assessed using Area Under the Receiver Operating Characteristic (AUROC) and Brier score; SHapley Additive exPlanations (SHAP) provided 28-day interpretability.

**Results:**

Of 8,048 patients, most were euglycemic (70–180 mg/dL) on day 1, although hyperglycemic excursions were frequent. Higher TWAG was associated with higher 28-day mortality: 140–179 mg/dL aHR 1.42 (95% CI, 1.25–1.62); ≥180 mg/dL aHR 1.41 (95% CI, 1.17–1.69). TWAG <100 mg/dL showed a nonsignificant trend toward higher survival. GlucoSurvAI achieved AUROC 0.967 (±0.008) with a low Brier score (~0.026). Adjusted SHAP analyses paralleled Cox results, identifying 100–139 mg/dL as the exposure range associated with decreased mortality, with risk increasing ≥140 mg/dL. First-day vasopressors and corticosteroids were also associated with higher mortality; insulin exposure marked higher risk after adjustment.

**Conclusions and Relevance:**

During the first ICU day, exposure-aware TWAG assessmentidentified a practical upper boundary near 140 mg/dL associated with higher 28-day mortality. An interpretable ensemble integrating TWAG, treatments, and physiology provided accurate first-day risk estimates, supporting risk-informed, individualized glycemic targets and earlier intervention in high-risk ICU patients.

## Introduction

Hyperglycemia is frequent in critical illness and associated with infection, organ dysfunction, and death. ^1,2^ Landmark evidence (NICE-SUGAR) demonstrated that intensive glucose targets (81–108 mg/dL) increased mortality and hypoglycemia compared with a conventional target ≤ 180 mg/dL, prompting a shift away from tight control in the ICU.^2,3^ Contemporary guidance recommends protocols that minimize hypoglycemia and generally initiate treatment for persistent hyperglycemia (≥ 180 mg/dL), but questions remain about optimal early targets and how best to summarize glycemia during the first ICU day.^3–6^

Most bedside assessments rely on single or sporadic glucose measurements; however, these may misclassify exposure when sampling is intermittent or clinically driven.^7–9^ Exposure-aware metrics, such as time-weighted average glucose (TWAG), integrate all measurements and their timing and may better reflect the early glycemic burden that is most relevant to short-term outcomes.^7,9,10^ Yet the relationship between first-day TWAG and 28-day mortality, and whether this information can be operationalized into accurate, interpretable first-day risk estimation, remains unclear.^7,9,11–13^

We aimed (1) to examine the association between first-day TWAG and 28-day all-cause mortality in a multicenter ICU cohort, and (2) to evaluate an interpretable ensemble model for early risk stratification that aligns model-based explanations with clinically adjusted time-to-event estimates. We hypothesized that higher first-day TWAG, particularly above a clinically relevant upper boundary, would be associated with increased 28-day mortality and that an interpretable model could capture this exposure–mortality gradient with strong discrimination and calibration.

## Methods

### Data Source

Our cohort was derived from Bridge2AI CHoRUS AIM-AHEAD60 project, a multicenter resource encompassing diverse ICU populations and clinical contexts from 13 U.S. hospitals. ^14,15^

### Participants

**Figure 1:**
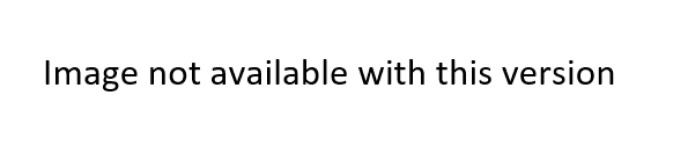
Cohort selection and glycemic bin classification. Flow diagram illustrating the derivation of the studycohort from 18,868 unique ICU patients. After applying inclusion criteria (age ≥18 years, >1 glucosemeasurement, and ICU stay >24 hours), 8,048 patients were retained for analysis. Patients werecategorized into end-of-day time-weighted average glucose (TWAG) bins using two approaches: (1)clinical glycemic states: hypoglycemic (N = 11), euglycemic (N = 7,306), and hyperglycemic (N = 731); and(2) numeric TWAG ranges: <100 mg/dL (N = 909), 100–139 mg/dL (N = 4,781), 140–179 mg/dL(N = 1,627), and ≥180 mg/dL (N = 731).

Eligible patients were aged 18–105 years, had a hospital length of stay ≥ 24 hours, and at least one glucose value recorded during the first ICU day. Among 18,868 unique ICU admissions, 8,048 from 7 of the 13 U.S. hopitals met inclusion criteria after restricting to the index ICU encounter. The participant flow diagram appears in Fig. 1.

### Exposures (Glycemic Categories)

Glycemic exposure during the first 24 hours of ICU care was summarized using time-weighted average (TWA) glucose. For clinical interpretability, patients were categorized as hypoglycemic (< 70 mg/dL), euglycemic (70–180 mg/dL), or hyperglycemic (> 180 mg/dL). In analyses examining dose–response, four TWAG bins were evaluated: <100, 100–139 (reference), 140–179, and ≥ 180 mg/dL.

### Outcomes

The primary outcome was all-cause mortality by 28 days; mortality by 180 days was a secondary outcome. Survival time was measured from the start of the first 24-hour ICU period. Kaplan–Meier estimates and multivariable time-to-event models were used as described below.

### Covariates and Potential Confounders

Prespecified covariates were selected based on clinical relevance and prior literature and included: diabetes status (prediabetes, diabetes), acute severity indicators (e.g., shock, vasopressor score), first-day treatment exposures (insulin, corticosteroids, vasopressors), cancer, and clinical site. To address potential bias from differential glucose monitoring, we adjusted for glucose monitoring intensity and cumulative exposure (e.g., first-day TWAG and number of glucose checks). Variable definitions and coding are detailed in eMethods.

### Statistical Analysis

Baseline characteristics are summarized overall and by outcome group (further discussed in eMethods in the Supplement). Survival across glycemic categories was evaluated using Kaplan–Meier curves and log-rank tests (descriptive). The primary inferential analysis used multivariable Cox proportional hazards models to estimate adjusted hazard ratios (aHRs) and 95% CIs for 28-day mortality across glucose categories (reference: 100–139 mg/dL). Models adjusted for the covariates described above, including clinical site, to mitigate confounding due to site-level practice variation; p-values are not emphasized in the narrative, consistent with our focus on aHRs and 95% CIs. Model assumptions and sensitivity analyses are described in eMethods in the Supplement.

To further contextualize glycemic exposure, we calculated restricted mean survival time (RMST) over a fixed 28-day horizon and compared RMST across the prespecified glucose bins (< 100 mg/dL, 100–139 mg/dL, 140–179 mg/dL, and ≥ 180 mg/dL) and across demographic strata (< 50, 50–64, 65–79, and ≥ 80 years; race; and ethnicity). RMST provides an interpretable, time-bounded measure of average survival and is robust when proportional hazards may not fully hold.^16–18^

### Prediction Models

For context and benchmarking, we compared traditional severity-score models (SOFA and APACHE II, fit as logistic regressions for 28-day mortality) with a stacked ensemble model (GlucoSurvAI) trained on first-day features. GlucoSurvAI integrates a survival-oriented neural network with gradient-boosted trees and a final meta-learner to yield calibrated short-term mortality predictions. Development procedures (preprocessing, cross-validation, hyperparameter tuning, calibration) and interpretability methods are provided in eMethods in the Supplement.

### Performance Assessment

Discrimination for binary 28-day mortality was summarized by AUROC, probability calibration by the Brier score. For time-to-event performance we report Harrell’s C-index. Threshold selection and classification metrics are described in eMethods and are not central to clinical interpretation of model comparisons in the main text.

### Software

Analyses were performed using Python-based tooling; package versions and code provenance are provided in eMethods.

### Reporting and Supplementary Materials

This manuscript adheres to STROBE and TRIPOD + AI recommendations; extended methods, definitions, and robustness checks appear in the eMethods/eTables/eFigures in the Supplement. JAMA Network encourages adherence to EQUATOR reporting guidance and use of the Supplement for technical detail.

### Ethics

Only de-identified data were used and institutional approvals aligned with participating sites’ data-use agreements. As part of Bridge2AI, this study was approved by each institution’s Institutional Review Board (IRB), and implied consent was applied because the dataset consisted of limited, de-identified records collected from participating sites and subsequently date-shifted at a central repository.

## Results

### Cohort selection and first-day glycemic distribution

Among 18,868 ICU admissions, 8,048 unique patients met inclusion criteria (≥ 1 glucose value and hospital length of stay ≥ 24 hours; Fig. 1). By clinical glycemic categories, most patients were euglycemic (70–180 mg/dL) on day 1, with smaller proportions classified as hypoglycemic (< 70 mg/dL) or hyperglycemic (> 180 mg/dL) (distribution in Table 1). When considering all measurements within the initial 24-hour window, 2,056 patients (25.5%) experienced at least one hyperglycemic episode, whereas 185 (2.3%) had at least one hypoglycemic episode; 7,068 patients (87.8%) remained entirely within the target range (eTable 1 in the Supplement). Of the 8,048 included patients, 158 lacked complete timestamp data for their initial glucose measurements, which prevented calculation of day-one TWAG; these patients were retained in categorical analyses and subsequently included in multivariable models using multiple imputation by chained equations (MICE) for missing time-weighted glucose values, as detailed in the eMethods in the Supplement.

### Baseline monitoring and site variation

Baseline monitoring and site differences are summarized in eTable 2 in the Supplement. Mortality differed across sites and was partly explained by case-mix and acuity: centers with higher SOFA/APACHE II burdens also had higher crude mortality. In adjusted models, we included clinical site to mitigate confounding by practice patterns and patient mix; core associations between TWAG bins and mortality were unchanged. Detailed site-level summaries and robustness checks are provided in eResults in the Supplement.

### Patient mortality by clinical glycemic category

**Figure 2:**
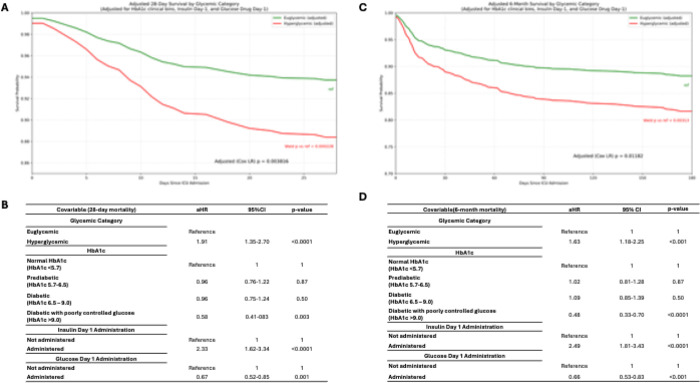
Adjusted Kaplan–Meier Survival Curves and Cox Hazard Ratios for Glycemic Categories and KeyCovariates **A)** Kaplan–Meier curves for 28-day survival stratified by glycemic category (euglycemic, hypoglycemic, hyperglycemic), adjusted for age, comorbidities, and treatment covariates. Hyperglycemiashows the steepest decline in survival, while hypoglycemia also confers elevated risk compared toeuglycemia. **B)** Kaplan–Meier curves for 180-day survival by glycemic category, illustrating persistentmortality risk associated with hyperglycemia and hypoglycemia beyond the acute phase. **C)** Survivalcurves stratified by HbA1c-defined chronic glycemic status (normal, prediabetes, diabetes, poorlycontrolled diabetes). Patients with poorly controlled diabetes (HbA1c >9%) exhibit distinct long-termsurvival patterns compared to normoglycemic individuals. **D)** Survival curves comparing patients withand without insulin administration on day 1 of ICU admission. Insulin exposure is associated with higheradjusted mortality risk, reflecting treatment complexity and underlying severity.

Kaplan–Meier curves showed significant differences in mortality across clinical glycemic categories (hypoglycemic < 70 mg/dL, euglycemic 70–180 mg/dL, hyperglycemic > 180 mg/dL), with higher mortality in hyperglycemia at both 28 days and 6 months (Fig. 2). In Cox models adjusted for HbA1c category and first-day insulin/glucose administration, hyperglycemia remained associated with increased mortality vs euglycemia (28-day aHR 1.91, 95% CI 1.35–2.70; 6-month aHR 1.63, 95% CI 1.18–2.25), while hypoglycemia was not significant after adjustment. Additional treatment and HbA1c associations are described in eResults in the Supplement.

### GlucoSurvAI shows superior predictive performance when compared to AI/ML models

**Figure 3:**
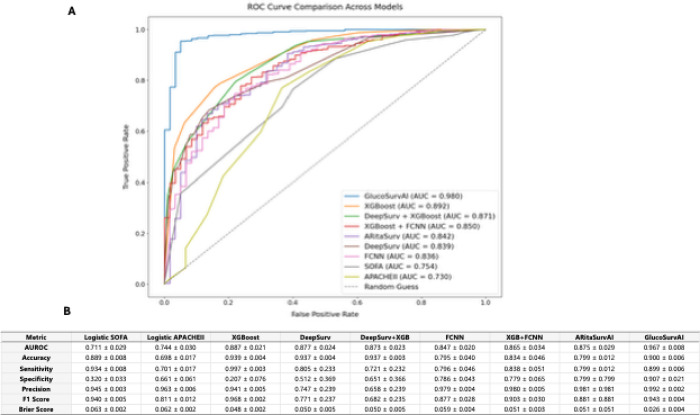
Comparative ROC Curves and Performance Metrics Across Candidate Models. **A)** Receiver OperatingCharacteristic (ROC) curves for all candidate models: SOFA, APACHE II, XGBoost, DeepSurv,DeepSurv + XGBoost, FCNN, XGBoost + FCNN, ARitASurv, and GlucoSurvAI, plotted against a “randomguess” diagonal baseline. GlucoSurvAI demonstrates the highest discrimination (AUROC ≈ 0.980),followed by XGBoost (≈ 0.894) and XGBoost + DeepSurv (≈ 0.892), while traditional severity scores (SOFAand APACHE II) show substantially lower performance (≈ 0.754 and ≈ 0.730, respectively). **B)** Summarytable of cross-validated performance metrics for each model, including AUROC, accuracy, sensitivity,specificity, precision, F1 score, and Brier score (mean ± SD). GlucoSurvAI achieved the best overallcalibration and discrimination (AUROC 0.967 ± 0.008; Brier score 0.026 ± 0.004), while maintainingbalanced sensitivity (0.899) and specificity (0.907), outperforming both single learners and otherensembles.

Figure 3B summarizes the performance of seven machine learning and deep learning models predicting 28-day mortality, evaluated across multiple metrics including AUROC, accuracy, sensitivity, specificity, precision, recall, F1 score, and Brier score.

GlucoSurvAI achieved the highest raw AUROC (0.967 ± 0.008) in ROC curve analysis (Fig. 3A), maintained strong accuracy (90%), balanced sensitivity (89.9%) and specificity (90.7%), and a high F1 score (94.3%), and a low Brier score (0.026). A schematic of GlucoSurvAI’s architecture is provided in eFigure 1 in the Supplement.

Additionally, site-level performance metrics showed that GlucoSurvAI AUROC values consistently exceeding 0.96 across major sites (Site 13: 0.988; Site 2: 0.980; Site 4: 0.964; Site 3: 0.963; Site 12: 0.975), coupled with high sensitivity (> 0.93) and strong negative predictive values (> 0.89) (eTable 3 in the Supplement).

### TWAG exposure bins: SHAP and adjusted hazards

**Figure 4:**
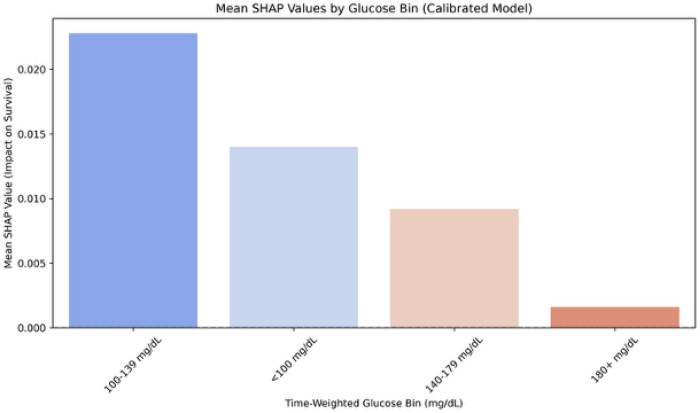
SHAP Analysis of Glycemic Bins and Survival Impact in GlucoSurvAI. SHAP (SHapley AdditiveexPlanations) summary plot illustrating the contribution of glucose bin features to survival predictionwithin the GlucoSurvAI ensemble. Glycemic exposures above 140 mg/dL were associated withprogressively higher mortality risk, with the strongest negative impact observed for glucose 180+ mg/dL.Conversely, the optimal survival range clustered between 100–139 mg/dL. Feature importance valuesreflect adjusted models controlling for multiple confounders, including diabetes status, septic orcardiogenic shock, first-day glucose, insulin and corticosteroid administration, vasopressor exposure,time weighted glucose and cancer status. This analysis underscores the additive prognostic value ofgranular glycemic bins beyond conventional thresholds.

**Figure 5:**
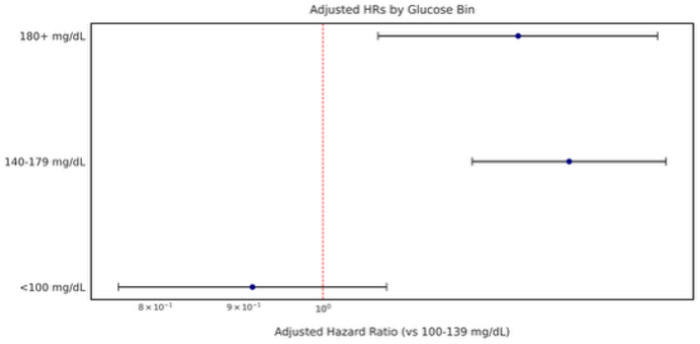
Forest Plot of Adjusted Hazard Ratios for Glycemic Bins. Forest plot displaying adjusted hazard ratios(aHR) and 95% confidence intervals for predefined glucose bins (<100 mg/dL, 100–139 mg/dL, 140–179 mg/dL, 180+ mg/dL) in relation to 28-day and 180-day mortality. Analyses were adjusted for keyconfounders, including diabetes status, HbA1c category, vasopressor exposure, corticosteroid use,glucose & insulin administration, septic or cardiogenic shock, and other clinical covariates. 100–139 mg/dL range served as a reference.

Model explanations from GlucoSurvAI, focused on 28-day survival and adjusted for diabetes/prediabetes, first-day insulin, glucose, corticosteroids, and vasopressors, shock, cancer, monitoring intensity, and clinical site indicated higher 28-day survival for 100–139 mg/dL, somewhat higher survival for < 100 mg/dL, and lower predicted survival for exposures ≥ 140 mg/dL (Fig. 4). These adjusted 28-day SHAP patterns aligned with adjusted 28-day GlucoSurvAI Cox models (Fig. 5, eTable 4A in the Supplement): relative to 100–139 mg/dL, mortality risk was higher for 140–179 mg/dL (aHR 1.42, 95% CI 1.25–1.62) and ≥ 180 mg/dL (aHR 1.41, 95% CI 1.17–1.69).

Beyond glycemic strata, several physiologic and clinical variables were independently associated with mortality after adjustment for confounders and for the reference glucose range (100–139 mg/dL). Among comorbidities, hypertension (aHR 0.76, 95% CI: 0.68–0.85) and COPD (aHR 0.84, 95% CI: 0.71–0.98) were associated with decreased mortality, whereas sepsis (aHR 1.28, 95% CI: 1.13–1.45) and brain injury (aHR 1.53, 95% CI: 1.22–1.93) markedly increased risk. Interestingly, psychological disorders (aHR 0.75, 95% CI: 0.67–0.84) and viral disease (aHR 0.69, 95% CI: 0.50–0.96) were associated with lower hazards, while organ failure involving heart, kidney, or liver showed modest decreased hazards (aHR 0.89, 95% CI: 0.79–0.99); which may indicate complex interactions with critical illness trajectories (eTable 4(A) and eFigure 2 in the Supplement).

When stratified by HbA1c category, risk patterns varied across glucose bins. In the ≥ 180 mg/dL group, patients with diabetes showed lower hazards compared with Normal HbA1c, whereas those with prediabetes exhibited increased risk. Diabetic patients with an end-of-day TWAG between 140–179 mg/dL had significantly decreased risk of mortality, while combinations within < 100 mg/dL and 100–139 mg/dL bins were largely neutral (eFigure 3 in the Supplement). Details on the effect of confounders may be found in eResults and eTable 4B in the Supplement.

### GlucoSurvAI Stratified Analysis of Glycemic Exposure by Demographic groups reveals at risk populations

Mortality was lowest in the 100–139 mg/dL range across all strata, with restricted mean survival time (RMST) approximating 27 days. Event rates increased sharply in the 140–179 mg/dL and ≥ 180 mg/dL bins, particularly among patients aged ≥ 80 years, where mortality exceeded 16% and RMST declined to ~ 24–25 days. Younger critically ill patients (< 50 years) also exhibited elevated risk at higher glucose levels, with event rates surpassing 25% in the 140–179 mg/dL and ≥ 180 mg/dL categories.

Across racial groups, White patients consistently demonstrated lower event rates and longer RMST within the optimal glycemic range, whereas Black and Other/Unknown race categories showed higher mortality in hyperglycemic strata. Ethnicity trends were less pronounced, but Hispanic patients in older age groups (≥ 65 years) exhibited increased mortality at glucose levels ≥ 140 mg/dL compared to non-Hispanic counterparts. These patterns were mirrored by higher Cox hazard scores and GlucoSurvAI-predicted mortality probabilities (eTable 5 in the Supplement).

We expanded our analyses to include major ICU admission reasons: injury/trauma, brain injury, neurological conditions, viral and bacterial infections, surgery, organ failure, sepsis, and psychological conditions, stratified by glycemic exposure and age (eTable 6 in the Supplement). Across all strata, sepsis and organ failure consistently exhibited the highest mortality, particularly in hyperglycemic patients aged ≥ 80 years, where event rates exceeded 18–22% and RMST declined to ~ 23–25 days. Elevated risk was also observed in younger patients (< 50 years) admitted for sepsis, with event rates approaching 19–23% in the 140–179 mg/dL and ≥ 180 mg/dL bins.

Conversely, patients admitted for psychological or trauma-related indications had lower mortality in the optimal glycemic range (100–139 mg/dL), with RMST near 27 days. Mortality risk increased substantially under hyperglycemia, with event rates rising to 7–11% in older adults, and up to 33% in younger trauma patients at ≥ 140 mg/dL. Neurologic and brain injury cases showed intermediate risk patterns, with mortality climbing sharply in the highest glucose strata, especially among the oldest patients. (eTable 6).

## Discussion

In this multicenter ICU cohort, we developed GlucoSurvAI, an interpretable, stacked survival model that integrates deep learning and gradient boosting to characterize first-day glycemia and predict 28-day mortality using early glycemic and physiologic data. Elevated first-day glucose exposure was associated with higher mortality, underscoring the importance of quantifying per-patient early glycemia rather than focusing solely on hypoglycemia avoidance. These findings align with evolving guidance that favors moderate glycemic targets and emphasizes minimizing both hyperglycemia and hypoglycemia in critical care.^4,12^

Most patients began ICU care within the euglycemic category by conventional clinical cut points, yet hyperglycemic excursions were common over the first 24 hours. We found that time-weighted average glucose (TWAG) offers a more comprehensive summary of cumulative exposure than single-point or short-window metrics, which can misestimate true burden when sampling is intermittent or clustered in time.^3,7,8^ By integrating every measurement and its duration, TWAG reduced misclassification evident with shorter windows, a pattern consistent with emerging reports that exposure-based metrics better reflect risk.^7,9^

Using prespecified TWAG exposure bins, both adjusted Cox models and adjusted SHAP explanations from GlucoSurvAI converged on 100–139 mg/dL as the range associated with higher 28-day survival, with higher risk beginning at 140 mg/dL. Relative to the 100–139 mg/dL reference, the 140–179 mg/dL and ≥ 180 mg/dL bins had higher adjusted hazards for 28-day mortality; <100 mg/dL trended toward higher survival but with wider uncertainty due to smaller numbers. The close alignment between adjusted SHAP (model-based explanations) and adjusted Cox (time-to-event estimates) increases confidence that this ~ 140 mg/dL inflection is a clinically meaningful signal in the initial 24 hours of an ICU admission.^7,19,20^

Interpreting glycemia in context is essential. In our adjusted models, first-day vasopressors and corticosteroids were associated with higher mortality, plausibly reflecting illness severity and treatment intensity, while insulin exposure also marked higher risk after adjustment. These associations highlight the interplay between hemodynamic support, pharmacologic therapy, and metabolic control, and reinforce that early glycemic exposure should be considered alongside acuity and therapeutic milieu when formulating risk-informed targets.^21–24^

Traditional ICU mortality prediction scores, such as SOFA and APACHE II, are well validated and have been widely adopted for decades.^25,26^ Moreover, both SOFA and APACHE scores provide a robust framework for assessing illness severity and evaluating organ function in critically ill patients.^26,27^ However, they were originally developed using older, often single-center cohorts, which may limit their ability to fully reflect contemporary patient complexity and evolving treatment practices.^25,26^ In this study, we leveraged a modern, multicenter ICU dataset to benchmark these established scores against advanced AI/ML models and novel glycemic indices derived from time-weighted glucose exposure. Our findings show that while APACHE II and SOFA maintain respectable predictive performance (AUROC = 0.749 and 0.754, respectively), they are substantially outperformed by GlucoSurvAI (AUROC = 0.976), which integrates dynamic glycemic metrics with physiological and comorbidity features. Additionally, compared with other AI/ML models, GlucoSurvAI avoids extreme trade-offs between precision and recall and offers practical advantages in speed, interpretability, and ease of deployment. These attributes make it a robust and well-rounded choice for survival prediction in this study.^28–30^

Beyond point estimates of accuracy, GlucoSurvAI preserved interpretability via SHAP, allowing clinicians to visualize how exposure bins and treatment covariates contribute to predicted risk at the bedside. Because the model relies on first-day information, it offers a practical path for early risk stratification that complements, and does not replace, established scores and clinical judgment.^30,31^

Stratified summaries suggested that the glycemia–survival gradient is context-dependent: it appeared steeper at higher exposure among older adults and in sepsis/organ-failure admissions, whereas some lower-acuity indications showed flatter gradients until exposure was markedly elevated. These patterns, together with the adjusted bin findings, support risk-informed targets rather than universal thresholds, consistent with literature showing heterogeneous glycemic risk across diagnoses and chronic glycemic status.^8,32^

In our adjusted analyses, patients with established diabetes (higher HbA1c) showed attenuated mortality risk in the upper TWAG bins, whereas those with prediabetes/normal HbA1c had higher risk at the same exposures. This pattern is consistent with prior work that indicate that acute stress hyperglycemia is more prognostic in patients without chronic hyperglycemia, while individuals with long-standing hyperglycemia may exhibit partial physiologic adaptation.^33–36^ Taken together, our results support individualized early glucose targets that account for baseline glycemic status (HbA1c) when interpreting first-day exposure.^8,37^

The present results reinforce a growing body of work linking higher early glycemic exposure and lower time-in-range with worse short-term outcomes in ICU populations.^7,9^ They also harmonize with the post–NICE-SUGAR shift away from intensive 80–110 mg/dL targets toward moderate ranges that reduce hypoglycemia risk while acknowledging the harm of sustained hyperglycemia.^4,38^ Importantly, our analysis quantifies an exposure-oriented signal during the first 24 hours and demonstrates how combining TWAG bins with an interpretable ensemble model can make this signal clinically actionable in real-time.

Strengths of our study include (1) a diverse, multisite cohort; (2) emphasis on exposure-aware metrics that reflect cumulative glycemia; (3) convergence between adjusted Cox and adjusted SHAP results; and (4) rigorous internal validation with discrimination and calibration reporting. Limitations include the retrospective design; variation in measurement frequency and modalities across sites; and the absence of standardized insulin/nutrition protocols, each of which may influence both exposure and outcomes. Although we adjusted for glucose monitoring intensity and included clinical sites to mitigate case-mix and practice differences, residual confounding remains possible. External validation and prospective evaluation, especially of model-informed glycemic management, are needed before clinical deployment (please see eAppendix).^12,39^

Collectively, our findings support maintaining early exposure in the 100–139 mg/dL range and avoiding sustained exposure ≥ 140 mg/dL during the first ICU day, while recognizing patient-specific factors, competing risks, and the real-world challenges of insulin titration. Pairing exposure-based metrics with interpretable model probabilities may help clinicians identify who is most likely to benefit from early titration and when the risk–benefit balance favors intervention, particularly in older adults and in high-acuity indications such as sepsis and organ failure.^7,9,37^

In conclusion, first-day glycemic exposure is common, measurable, and actionable. TWAG offers a robust summary of early glycemia, and both adjusted Cox and adjusted SHAP indicate 100–139 mg/dL is associated with higher 28-day survival, with higher risk beginning at 140 mg/dL. GlucoSurvAI serves as an interpretable ensemble model that leverages these exposure-aware metrics enables early, individualized risk stratification and provides a foundation for prospective, risk-informed glycemic management in critical care.^12,37^

## Supplementary Material

This is a list of supplementary fi les associated with this preprint. Click to download.

- SupplementalJOO.docx
- SupplementaleTablesJOO.xlsx
- eAppendixJOO.docx

## Key Points

### Question:

Among adults admitted to the ICU, is first-day glycemic exposure associated with 28-day mortality, and can an interpretable AI/ML model provide accurate early risk stratification?

### Findings:

In a multicenter cohort of 8,048 ICU patients, higher first-day time-weighted average glucose (TWAG) showed a strong dose–response relationship with 28-day mortality, with a clear inflection at ≥140 mg/dL. GlucoSurvAI accurately captured this exposure–mortality gradient and outperformed traditional severity scores.

### Meaning:

Maintaining early glycemic exposure near 100–139 mg/dL may be associated with lower mortality.
